# Conformational Itinerary of Sucrose During Hydrolysis by Retaining Amylosucrase

**DOI:** 10.3389/fchem.2019.00269

**Published:** 2019-04-30

**Authors:** Santiago Alonso-Gil, Joan Coines, Isabelle André, Carme Rovira

**Affiliations:** ^1^Departament de Quimica Inorgànica i Orgànica (Secció de Química Orgànica) and Institut de Quimica Teòrica i Computacional, Universitat de Barcelona, Martí i Franquès 1, Barcelona, Spain; ^2^Laboratoire d'Ingénierie des Systèmes Biologiques et des Procédés, LISBP, Université de Toulouse, CNRS, INRA, INSA, Toulouse, France; ^3^Institució Catalana de Recerca i Estudis Avançats (ICREA), Barcelona, Spain

**Keywords:** glycoside hydrolases, quantum mechanics/molecular mechanics, double-displacement reaction, *ab initi*o molecular dynamics, metadynamics, conformational analysis

## Abstract

By means of QM(DFT)/MM metadynamics we have unraveled the hydrolytic reaction mechanism of *Neisseria polysaccharea* amylosucrase (*Np*AS), a member of GH13 family. Our results provide an atomistic picture of the active site reorganization along the catalytic double-displacement reaction, clarifying whether the glycosyl-enzyme reaction intermediate features an α-glucosyl unit in an undistorted ^4^*C*_1_ conformation, as inferred from structural studies, or a distorted ^1^*S*_3_-like conformation, as expected from mechanistic analysis of glycoside hydrolases (GHs). We show that, even though the first step of the reaction (glycosylation) results in a ^4^*C*_1_ conformation, the α-glucosyl unit undergoes an easy conformational change toward a distorted conformation as the active site preorganizes for the forthcoming reaction step (deglycosylation), in which an acceptor molecule, i.e., a water molecule for the hydrolytic reaction, performs a nucleophilic attack on the anomeric carbon. The two conformations (^4^*C*_1_ ad *E*_3_) can be viewed as two different states of the glycosyl-enzyme intermediate (GEI), but only the *E*_3_ state is preactivated for catalysis. These results are consistent with the general conformational itinerary observed for α-glucosidases.

## Introduction

Glycoside hydrolases (GHs) are enzymes responsible for the degradation or hydrolysis of glycosidic bonds in carbohydrates. GHs are of fundamental interest in glycobiology and glycomics (Gamblin et al., 2009) since they are responsible for the modification of polysaccharides and glycoconjugates involved in numerous biological processes such as cell-cell recognition and polysaccharide degradation for biofuel processing (Pauly and Keegstra, 2008). These enzymes also provide paradigms for enzymatic catalysis that extend beyond the bounds of carbohydrate chemistry (Koshland, 1953; Davies et al., 2012). GHs are systematically classified in 156 families (by March 2019) according to their sequence similarity (http://www.cazy.org) (Cantarel et al., 2009). Enzymes from the same family usually share the same catalytic mechanism, either with retention or inversion of the anomeric configuration (Figure S1), in which an oxocarbenium ion-like transition state is formed (Rye and Withers, 2000; Zechel and Withers, 2000). Enzymes from the same family also share the same *conformational itinerary* of the saccharide unit at the *-1* subsite (hereafter named *-1* sugar), in which this saccharide unit changes conformation following a straight line or a equatorial one in Stoddart's diagram (Figure S2), either the one representing the Northern of Southern projection of the puckering sphere (Iglesias-Fernandez et al., 2015). Because there are a few conformations that satisfy the stereoelectronic requirements for a stable oxocarbenium ion (^4^*H*_3_,^3^*H*_4_, *B*_2,5_,^2,5^*B*, ^3^*E, E*_3_, ^4^*E*, and *E*_4_) the number of itineraries is limited (Speciale et al., 2014; Ardèvol and Rovira, 2015). However, there are several GH families for which the itinerary has not been proven yet or it is controversial.

Members of GH13 family are retaining GHs that catalyze the cleavage of the α-glucosidic linkages, such as α-amylase, responsible for starch and amylose degradation. This family also gathers unique enzymes called amylosucrases that catalyze from sole sucrose the synthesis of α-D-glucopyranosyl homopolymers and oligomers accompanied with limited sucrose hydrolysis (Albenne et al., 2004). These enzymes are thus considered to be both glycoside hydrolases and transglucosylases. The substrate conformational itinerary from the Michaelis complex (MC, Figure S1) to the covalent glycosyl-enzyme intermediate (GEI), is expected to be identical.

Several X-Ray structures on GH13 enzymes have demonstrated that the conformation of the *-1* sugar ring in the MC is ^4^*C*_1_ (Fujimoto et al., 1998; Mirza et al., 2001; Skov et al., 2002). The lack of distortion in the conformation, unlike many GHs, is because the α-stereochemistry at the anomeric carbon assures that the leaving group (+*1* sugar) is in a reactive orientation for the S_N_2 reaction to take place, unlike e.g., β-glucosidases (Biarnés et al., 2006, 2011). In fact, α-glucosidases have been suggested to follow a ^4^*C*_1_ → [^4^*H*_3_]^‡^ → ^1^*S*_3_ itinerary, which is the opposite as the ^1^*S*_3_ → [^4^*H*_3_]^‡^ → ^4^*C*_1_ for β-glucosidases (Davies et al., 2012). These differences are being exploited in the field of inhibitor design (Beenakker et al., 2017).

**Figure 1:**
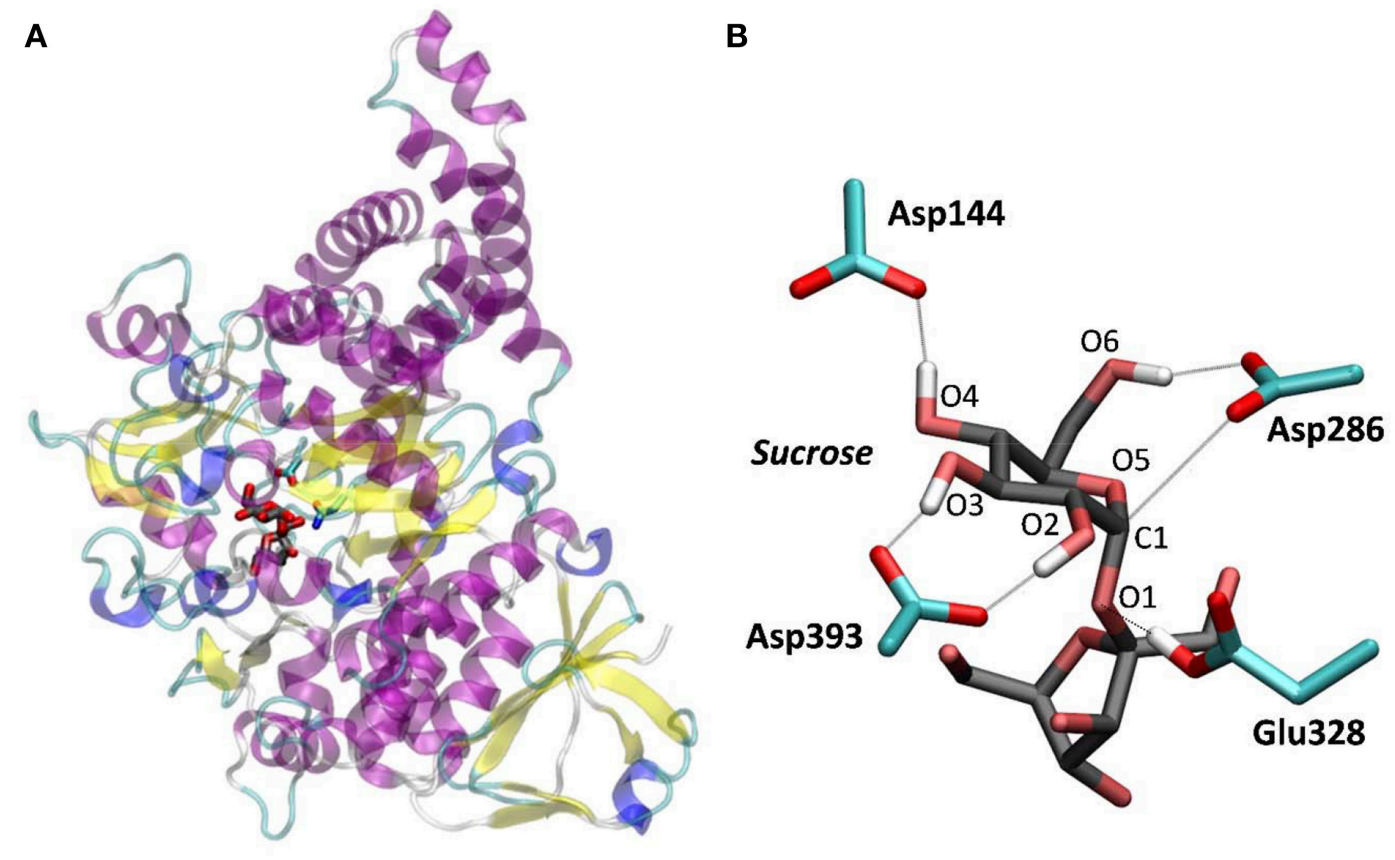
*Np*AS in complex with sucrose. **(A)** Crystal structure from PDB 1JGI. **(B)** QM/MM optimized structure of the active site (the Glu328Gln mutation of the crystal structure was reverted).

Concerning the glycosyl-enzyme covalent intermediate (GEI) of the reaction, structural analyses of GH13 amylosucrase show that the *-1* sugar bears a ^4^*C*_1_ conformation (Jensen et al., 2004). In the case of α-amylase, the GEI trapped using sugar analogs exhibits a similar ^4^*C*_1_ conformation (Zhang et al., 2009; Caner et al., 2016). QM/MM calculations of human pancreatic α-amylase captured the relevant role of the catalytic residues during the hydrolysis mechanism (Pinto et al., 2015), but the conformational itinerary of the *-1* sugar was not investigated. On the other hand, the GEI of *Bifidobacterium adolescentis* sucrose phosphorylase, a GH13 enzyme, shows the *-1* sugar distorted in a ^1^*S*_3_ conformation, suggesting a completely different conformational pathway (^4^*C*_1_ → TS → ^1^*S*_3_) (Mirza et al., 2006). Being both enzymes from the same family and acting over the same substrate, this discrepancy is puzzling. The ^4^*C*_1_ conformation of the GEI is also in contrast with the irreversible cyclosulphate inhibitors of α-glucosidases from other GH families, which show an unambiguous ^1^*S*_3_ conformation of the GEI analog (Artola et al., 2017). To solve this conundrum, we here uncover the conformational itinerary of the α-glucosyl unit of *Neisseria polysaccharea* amylosucrase (*Np*AS), a member of family GH13 for which a structure of the Michaelis complex with the natural substrate (sucrose) is available (Figure 1), during catalysis. Our simulations, performed by means of QM/MM metadynamics methods, show that the GEI can exhibit both a relaxed ^4^*C*_1_ conformation or a distorted *E*_3_-like conformation of the *-1* sugar, depending on whether the catalytic water is properly placed and oriented for catalysis or it is on its way in.

## Results

### Michaelis Complex Structure

To model the Michaelis complex of *Np*AS, we reverted the Glu328Gln mutation of the X-ray structure of the *Np*AS-sucrose complex and performed classical MD simulations. The conformation of the α-glucosyl ring at the *-1* sugar changed from ^4^*C*_1_ to *B*_3,O_ during the first 6 ns of the MD simulation, due to an increase in the distance between the nucleophile and the anomeric carbon, but returned to ^4^*C*_1_ during the remaining 8 ns, indicating that it is probably the most stable state. The ^4^*C*_1_ conformation was maintained during the subsequent QM/MM MD equilibration.

**Figure 2:**
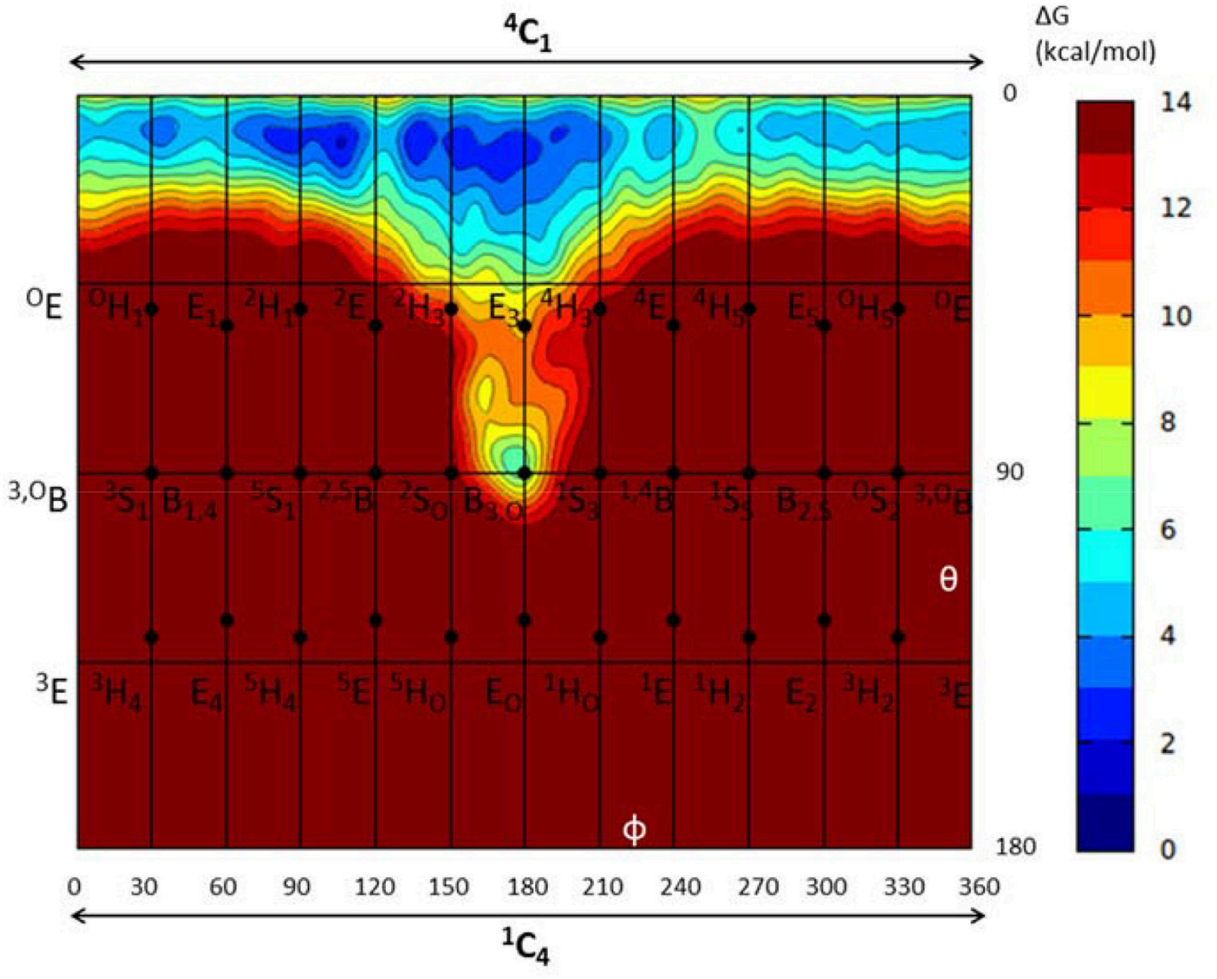
Conformational free energy landscape (FEL, in a Mercator representation) of the *-1* sugar (α-glucosyl) of *Np*AS in complex with sucrose. Contour lines at 1 kcal/mol.

To further ascertain which is the most favored conformation, we computed the conformational free energy landscape (FEL) of the α-glucosyl ring at the *-1* subsite by QM/MM metadynamics, using the Cremer-Pople puckering coordinates θ/φ as collective variables. This is a well-tested approach that we have used with success to analyse the conformation of carbohydrates in isolation or in the active sites of GHs (Biarnés et al., 2007; Alonso-Gil et al., 2017). The computed FEL, shown in Figure 2, confirms that the most stable conformation is ^4^*C*_1_, with a secondary minimum (4.7 kcal/mol above in free energy) corresponding to a *B*_3,O_ distorted conformation. The most relevant hydrogen bond interactions involving the *-1* sugar for the most stable ^4^*C*_1_ conformer are listed in Table 1 (see atom labeling in Figure 1B). This conformer shows a longer glycosidic bond distance (C1-O1 = 1.44 Å) compared to *B*_3,O_, (1.42 Å) and the C1-O5 bond is shorter by 0.03 Å. Notably, the distance between the nucleophile oxygen (O_Asp393_) and the anomeric carbon (C1) is shorter when the sugar is not distorted, with values of 3.10 Å (^4^*C*_1_) and 3.30 Å (*B*_3,O_), which would facilitate the nucleophilic attack by Asp286. Clearly, only the ^4^*C*_1_ conformation of the substrate is preactivated for catalysis.

**Table 1:** Calculated values of the most important interactions between the *-1* α-glucosyl residue and the enzyme in the Michaelis complex (energies in kcal/mol, distances in Å).

**Conformation**	**^**4**^*C*_**1**_**	***B*_**3, O**_**
φ, θ	136°, 10°	176°, 87°
ΔG	0.0	4.7
C1 – O1	1.44 ± 0.05	1.42 ± 0.04
C1 – O5	1.42 ± 0.04	1.45 ± 0.04
2-OH – O_Asp393_	1.51 ± 0.10	1.54 ± 0.09
3-OH – O'_Asp393_	1.60 ± 0.16	1.66 ± 0.16
4-OH – O_Asp144_	1.49 ± 0.09	1.50 ± 0.09
6-OH – O'_Asp286_	1.63 ± 0.24	1.66 ± 0.16
C1 – O_Asp286_	3.10 ± 0.12	3.30 ± 0.16

### Glycosylation Reaction

The simulation of the first step of the double displacement reaction (glycosylation) was initiated from a snapshot of the global minimum (^4^*C*_1_ conformation) of the conformational FEL of the Michaelis complex. Three collective variables were used to drive the glycosylation reaction (Figure S3 and Methods section), representing the proton transfer (CV1), the nucleophilic attack (CV2) and the glycosidic bond cleavage (CV3).

**Figure 3:**
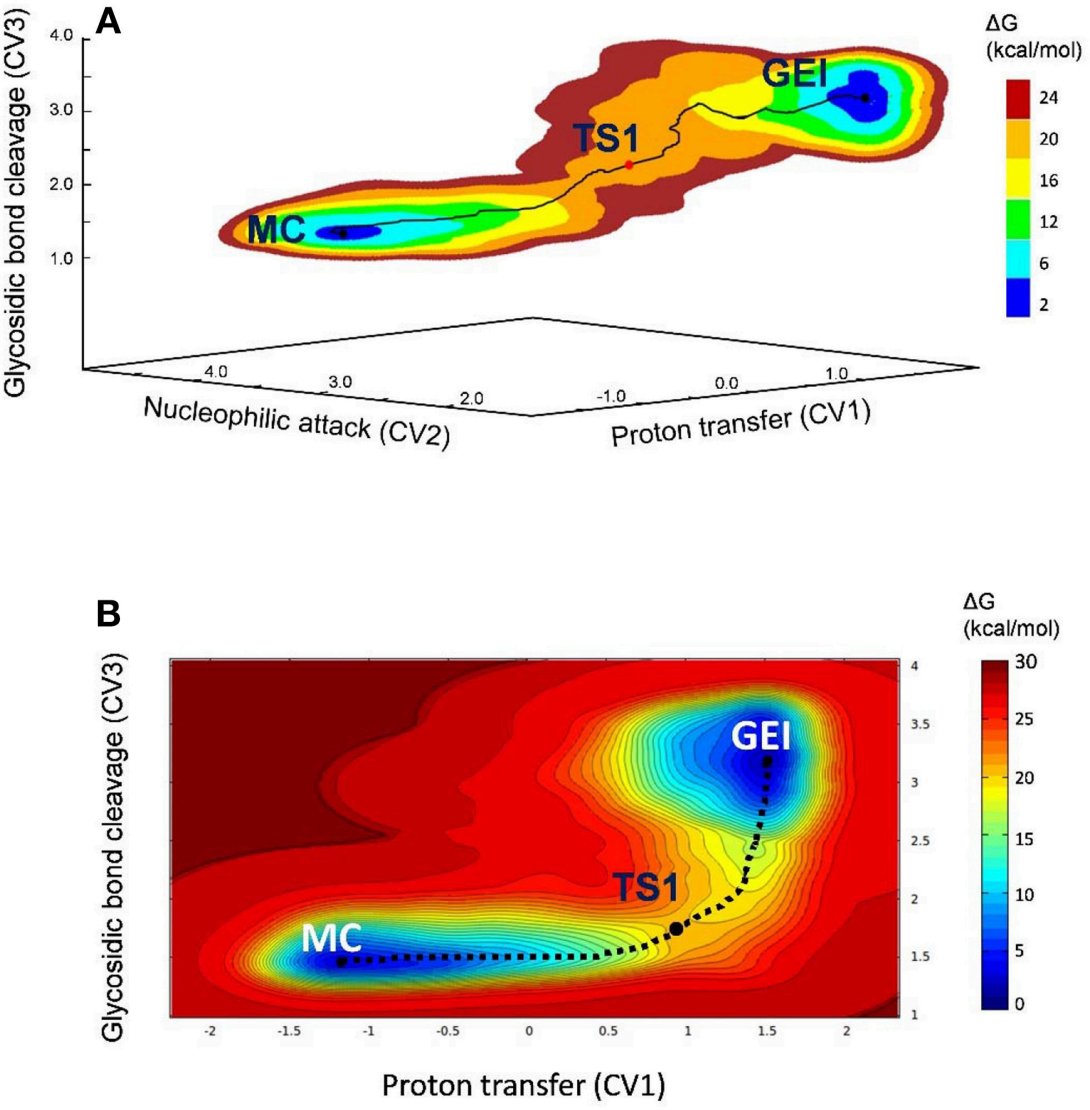
**(A)** Free energy landscape of the glycosylation reaction, obtained from QM/MM metadynamics simulations with three CVs. **(B)** Two-dimensional projection of the FEL into the collective variables CV1 and CV3.

The free energy landscape reconstructed from the metadynamics simulation (reaction FEL) is shown in Figure 3A (for further detail, Figure 3B shows a two-dimensional projection). The FEL exhibits two clear minima in opposite regions: reactants (Michaelis complex, MC) and products (glycosyl-enzyme intermediate, GEI), separated by a transition state (TS1). The rate-limiting step of *Np*AS is not known but we can assume it is the glycosylation step, as in most GHs acting on substrates with a sugar aglycone (Li et al., 2001). Under this assumption, the reaction free energy barrier (17.2 kcal/mol) is in very good agreement with the experimental value of 17.9 kcal/mol estimated from the room temperature rate constant (Potocki de Montalk et al., 2000).

**Figure 4:**
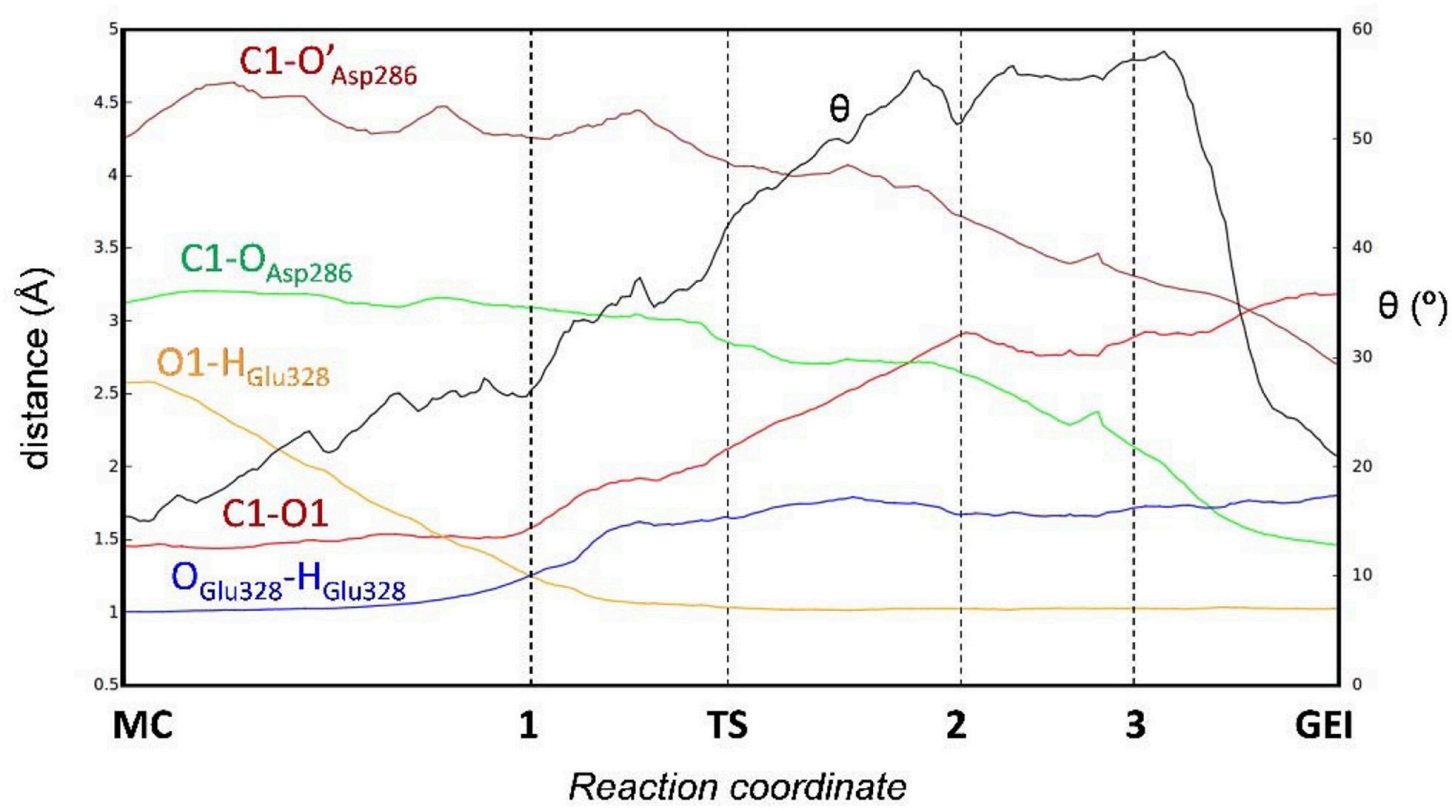
Evolution of relevant active site distances during the glycosylation reaction catalyzed by *Np*AS.

**Figure 5:**
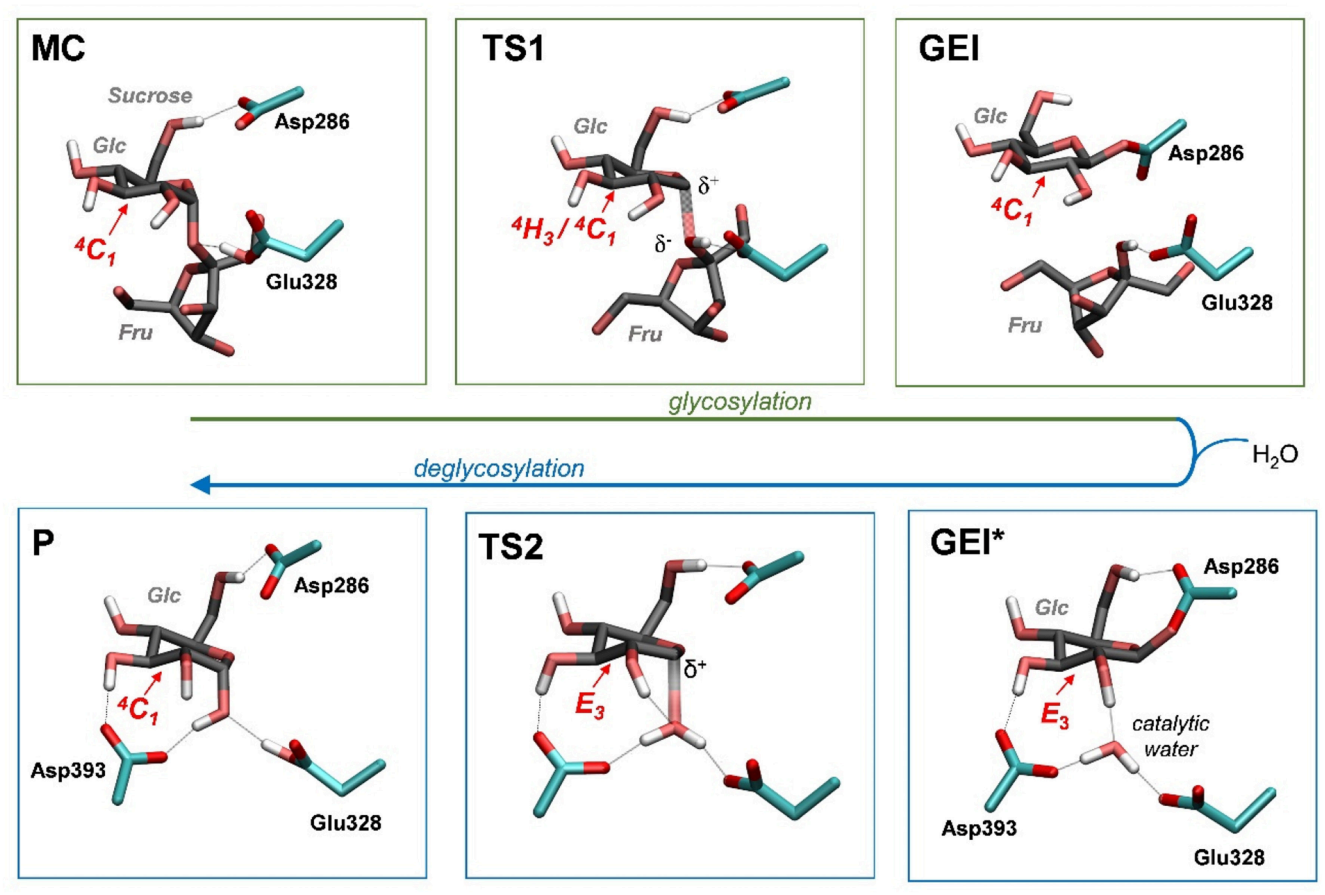
Reaction mechanism of *Np*AS obtained in this work (MC, Michaelis complex; TS, transition state; GEI, glycosyl-enzyme intermediate; P, reaction products). Hydrogen atoms have been also omitted, except the ones attached to heteroatoms. For the sake of clarity, the leaving β-fructose molecule has been omitted from the deglycosylation reaction (bottom panels).

Figure 4 and Table 2 show the evolution of the main catalytic distances along the minimum energy pathway and the structures of the active site at the stationary states are represented in Figure 5 (top panels). The glycosylation reaction begins with the approach of Glu328 to the glycosidic oxygen. The distance between the Glu328 acid/base proton and the glycosidic oxygen (O1-H_Glu328_) quickly decreases from 2.5 to 1.2 Å. Afterwards (**1** in Figure 4), the acid/base residue transfers the proton to the glycosidic oxygen and the glycosidic bond (C1-O1) starts to increase. Protonation of the glycosidic oxygen takes place in the *anti*-configuration with respect to the C1-O5 bond, as common in α-glycosidases (Alonso-Gil et al., 2017). At the reaction transition state (TS1), the glycosidic bond is partially broken (1.98 Å) but the nucleophile has not yet started to attack the anomeric carbon (2.98 Å). Therefore, the reaction follows a dissociative mechanism and can be described as D_N_A_N_ (Guthrie and Jencks, 1989; Schramm and Shi, 2001). Afterwards, the distance between the nucleophilic oxygen of Asp286 and the anomeric carbon decreases and the glycosyl-enzyme covalent bond forms. As it was found for endo-β-glucanase (Biarnés et al., 2011), the maximum oxocarbenium ion character does not occur at the TS but later on the reaction pathway (**2** in Figure 4). At this point, both the nucleophile and the leaving group are well separated from the C1 atom and its charge is higher than the one at the TS (by 0.05 electrons, Figure S4). The calculations thus reveal that the glycosylation reaction features an early TS with respect to charge development. The glycosyl-enzyme intermediate is almost formed at **3** (C1-O_Asp286_ = 2.14 Å). Finally, the hydrogen bond interaction between the free oxygen of Asp286 (O') and the 6-OH breaks and Asp286 rotates around the covalent bond with the *-1* sugar, which collapses to an undistorted ^4^*C*_1_ conformation (GEI).

**Table 2:** Calculated values of the most relevant catalytic distances (in Å) and puckering coordinates (in degrees) and their standard deviations along the glycosylation minimum energy pathway.

	**ΔG**	**C1 – O_**Asp286**_**	**C1 – O1**	**O1 – H_**Glu328**_**	**O_**Glu328**_ – H_**Glu328**_**	**θ**	**Conformation**
MC	0.0	3.13 ± 0.09	1.46 ± 0.11	2.58 ± 0.07	1.00 ± 0.03	16 ± 7	^4^*C*_1_
1	14.2	3.08 ± 0.10	1.58 ± 0.12	1.25 ± 0.08	1.25 ± 0.08	27 ± 8	^4^*C*_1_
TS1	17.3	2.98 ± 0.11	1.98 ± 0.10	1.05 ± 0.05	1.61 ± 0.12	37 ± 8	^4^*H*_3_/^4^*C*_1_
2	14.4	2.72 ± 0.14	2.79 ± 0.12	1.02 ± 0.04	1.72 ± 0.13	55 ± 8	*E*_3_
3	11.7	2.14 ± 0.17	2.89 ± 0.12	1.02 ± 0.04	1.71 ± 0.12	57 ± 8	^4^*H*_3_
GEI	−1.5	1.46 ± 0.05	3.18 ± 0.11	1.02 ± 0.04	1.78 ± 0.13	21 ± 8	^4^*C*_1_

**Figure 6:**
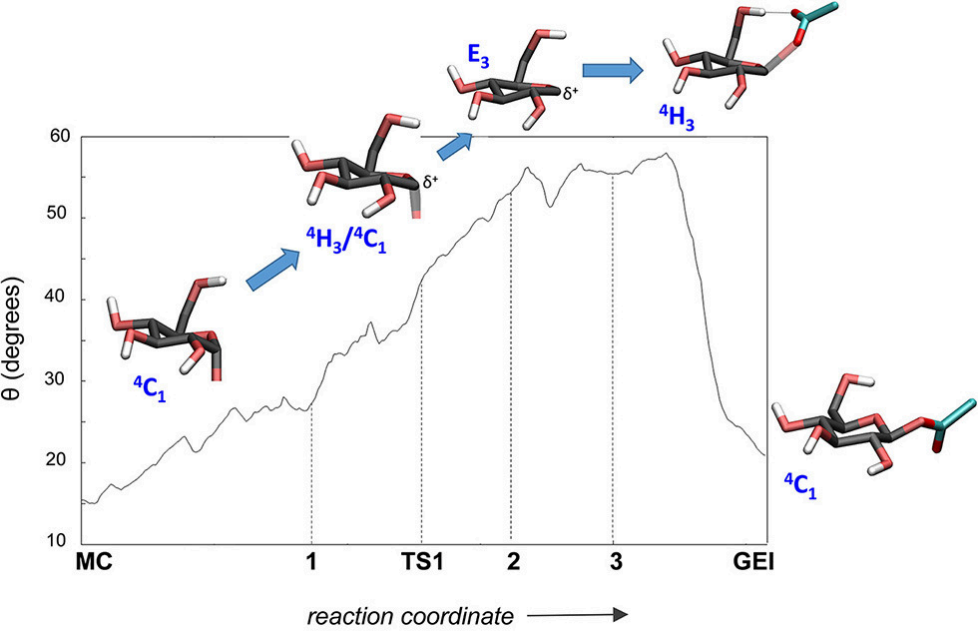
Evolution of the conformation of the *-1* sugar (α-glucosyl residue) during the glycosylation reaction.

It is interesting to analyse in detail the conformation of the *-1* sugar during the reaction, in relation with the hydrogen bond between the nucleophile residue (Asp286) and the 6-OH. The evolution of the sugar conformation during the glycosylation reaction, defined by the θ puckering coordinate, is shown in Figure 6. From the MC to the TS, the conformation evolves from ^4^*C*_1_ to ^4^*H*_3_/^4^*C*_1_. Afterwards, the sugar adopts an *E*_3_ envelope conformation (**2**), followed by a ^4^*H*_3_ half-chair (**3**). At this point, a sudden conformational change toward ^4^*C*_1_ takes place (θ changes from 58° to 20°), concomitant with rotation of Asp286 around the glycosyl-enzyme bond, and the disruption of the 6-OH hydrogen bond interaction. The covalent intermediate is already formed before the rotation and the two configurations of the GEI (with and without hydrogen bond with the 6-OH) are close in energy. However, only the one without the hydrogen bond, and thus with a ^4^*C*_1_ conformation, corresponds to a minimum on the FEL of Figure 3. Thus, the conformation of the GEI after the first step of the double displacement reaction is ^4^*C*_1_. In other words, the conformational itinerary is in principle cyclic, i.e., it starts and ends up in the same conformation (^4^*C*_1_ → [^4^*H*_3_/*E*_3_]^‡^ → ^4^*C*_1_). This is in agreement with the conformations of the experimental structures of the MC and GEI of *Np*AS (both in ^4^*C*_1_ conformation) (Mirza et al., 2001; Skov et al., 2002; Jensen et al., 2004). However, the computed itinerary differs from the expected one for α-glucosidases, for which a distorted GEI is expected (^4^*C*_1_ → [^4^*H*_3_]^‡^ → ^1^*S*_3_) (Davies et al., 2012; Speciale et al., 2014). As we will see in the next section, both views can be reconciled if we consider the reorganization of the active site required to start the deglycosylation reaction.

### Deglycosylation Reaction

**Figure 7:**
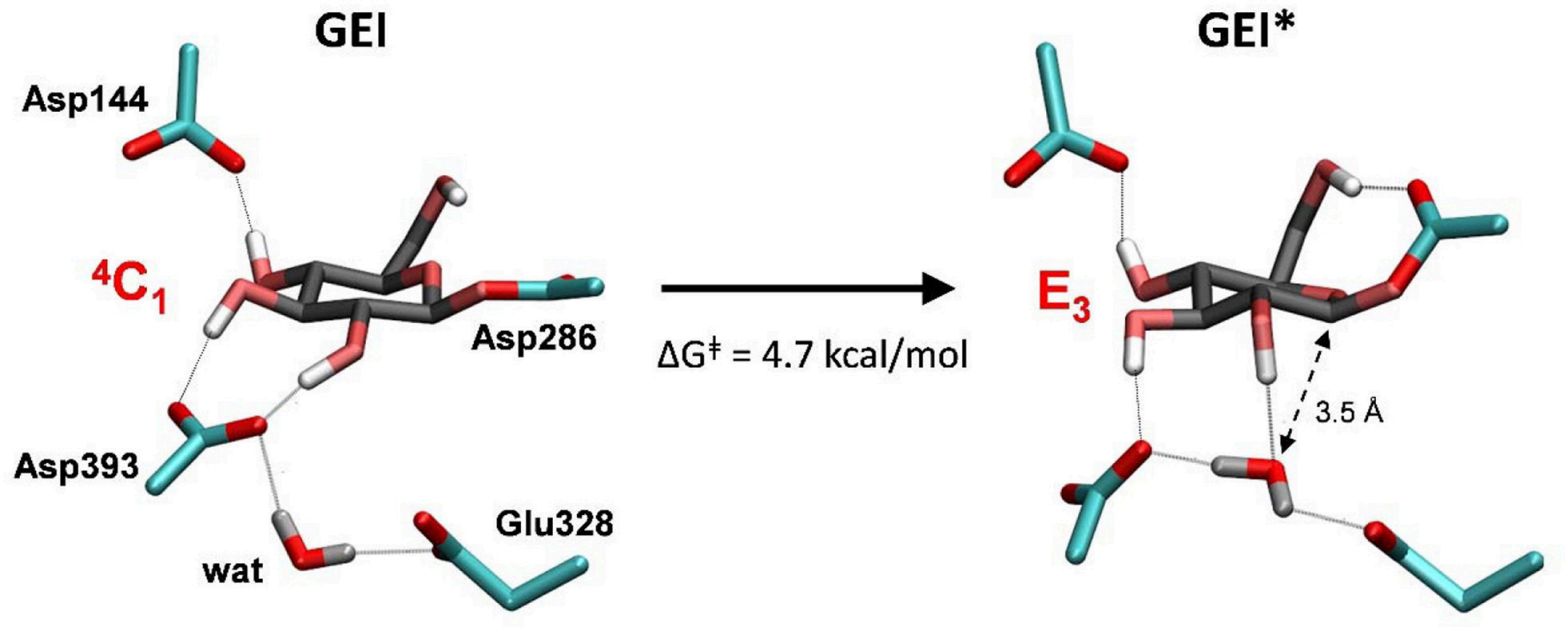
Reorganization of the active site of *Np*AS in the GEI state as the catalytic water approaches the anomeric carbon. The leaving β-fructose (product of the glycosylation reaction) is not shown for clarity.

As shown above, the glycosylation reaction leads to an active site configuration in which the *-1* sugar is in a ^4^*C*_1_ conformation. This configuration is not suitable for the second step of the double displacement reaction for two main reasons. First, the leaving group (C1-O_Asp286_) is not in axially oriented, which makes the S_N_2 reaction unfavorable. Second, there is no water molecule in the vicinity of the anomeric carbon. The nearest water molecule remains at ~6 Å from the anomeric carbon in the simulation (Figure 7, left panel), forming hydrogen bond interactions with the acid/base residue (Glu328) and an aspartate residue (Asp393). This water molecule is the best candidate to act as a nucleophile in the deglycosylation process, but it is still too far and not well oriented to attack the anomeric carbon. Most likely, there is an energy barrier to bring the water molecule to a reactive configuration.

To obtain the reactive configuration for the deglycosylation reaction, we selected the above water molecule and approached it to the anomeric carbon with the metadynamics algorithm using the O_water_-C1 distance as collective variable. In order to allow the C1-Asp286 bond to adopt an axial orientation (necessary for the water nucleophilic attack), a second collective variable that accounts for the hydrogen bond between Asp286 and the 6-OH was used. This simulation facilitated the identification of a configuration of the glycosyl-enzyme intermediate in which the water molecule is at ≈ 3.5 Å from C1 (Figure 7, right panel). The position of the water molecule is stabilized by hydrogen bond interactions with the 2-OH of the *-1* sugar, Asp393, and the Glu328 catalytic residue. The new configuration, hereafter named GEI^*^, is separated from the initial GEI by a small free energy barrier (4.7 kcal/mol; Figure S5). Remarkably, the *-1* sugar at GEI^*^ is preactivated for catalysis, as it exhibits a distorted conformation (*E*_3_) with a pseudo-axial orientation of the leaving group. Moreover, the water molecule is well oriented for nucleophilic attack, as it has the oxygen atom lone pairs pointing toward the anomeric carbon. Therefore, this configuration is the one we chose to start the modeling of the deglycosylation reaction. Interestingly, a distorted conformation of the GEI of lysozyme, a family 22 β-GH, was recently described by the Mulholland group based on QM(SCC-DFTB)/MM calculations (Limb et al., 2019). However, the GEI^*^ states found here differs from the one of (Limb et al., 2019) in that the nucleophile carboxylate group exhibits the usual syn configuration with respect to the C1-O_Asp286_ bond (τ_C1−O−C−O_ ≈ 0°). Differences in the substrate, active site and reaction stereochemistry might be the reason of the discrepancy.

**Figure 8:**
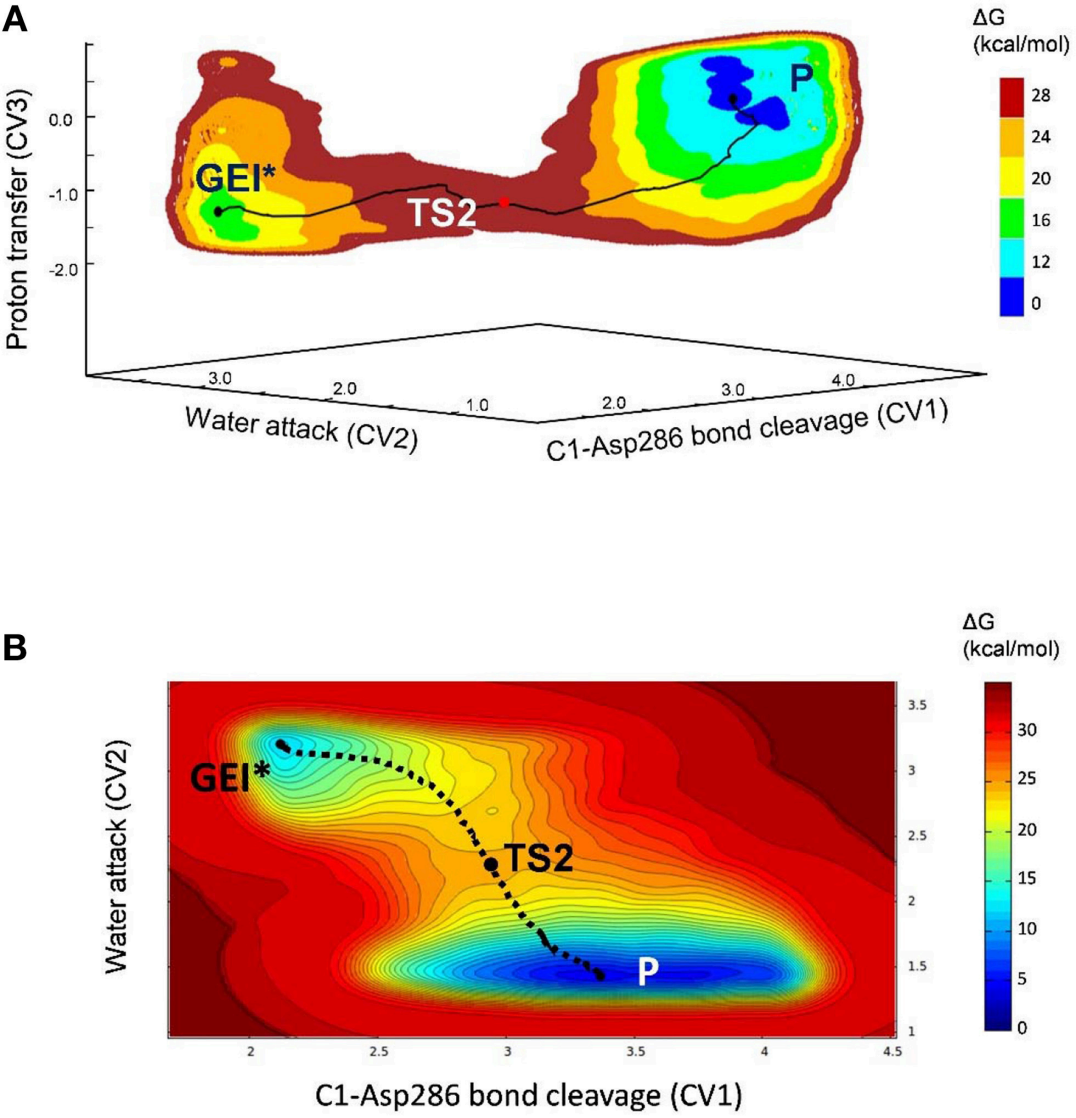
**(A)** Free energy landscape (FEL) of the deglycosylation reaction, obtained from QM/MM metadynamics simulations with three CVs. **(B)** Two-dimensional projection of the FEL into the collective variables CV1 and CV2.

The deglycosylation reaction was modeled using three collective variables that take into account the cleavage of the glycosyl-enzyme covalent bond (C1-O_Asp286_), the attack of the water oxygen to the anomeric carbon (C1-O_wat_), and the deprototonation of the water molecule by Glu328. The FEL reconstructed from the metadynamics simulation, shown in Figure 8, shows two minima corresponding to the activated glycosyl-enzyme intermediate (GEI^*^) and the hydrolysis products (P), which are 7.6 kcal/mol more stable. The transition state (TS2) is 13.3 kcal/mol higher in energy with respect to the GEI^*^ state, consistent with deglycosylation not being rate limiting.

**Figure 9:**
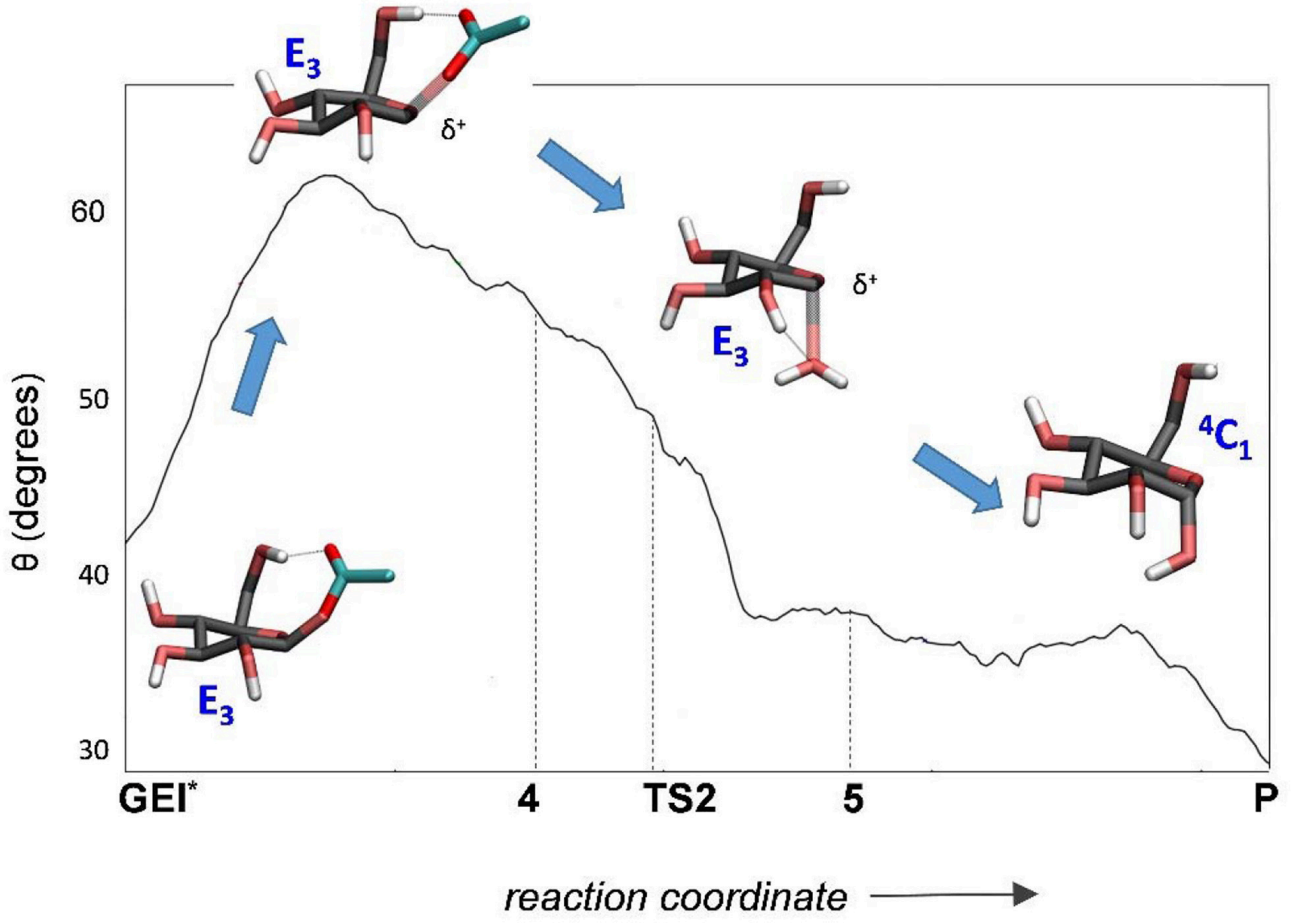
Evolution of the conformation of the *-1* sugar (α-glucosyl residue) during the deglycosylation reaction.

The structure of the active site and the *-1* sugar conformation along the deglycosylation reaction pathway is shown in Figure 5 (bottom panels) and Figure 9, whereas Table 3 lists the evolution of the most important distances. The deglycosylation reaction begins by cleavage of the glycosyl-Asp286 bond, followed by attack of the water molecule on the anomeric carbon, while the water forms a tight hydrogen bond with the acid/base residue (Glu328). The system overcomes the transition state (TS2) and the conformation of the *-1* sugar changes to an *E*_3_ envelope. Afterwards, the Glu328 abstracts a proton from the catalytic water, the covalent bond between the water molecule and the anomeric carbon forms and the -1 sugar adopts a ^4^*C*_1_ conformation. Therefore, the conformational itinerary of the deglycosylation reaction of *Np*AS can be described as *E*_3_ → [*E*_3_]^‡^ → ^4^*C*_1_.

**Table 3:** Calculated values of the most relevant catalytic distances (in Å) and their standard deviations along the deglycosylation minimum energy pathway.

	**ΔG**	**C1 – O_**Asp286**_**	**C1 – O_**wat**_**	**H-O_**wat**_**	**H'_**wat**_-O_**Glu328**_**	**θ**	**Conformation**
GEI*	0.0	2.14 ± 0.09	3.19 ± 0.11	1.02 ± 0.04	1.65 ± 0.10	42 ± 11	*E*_3_
4	11.0	2.81 ± 0.12	2.50 ± 0.12	1.06 ± 0.06	1.48 ± 0.10	52 ± 9	*E*_3_
TS2	13.3	2.81 ± 0.12	2.23 ± 0.12	1.05 ± 0.05	1.53 ± 0.11	48 ± 7	*E*_3_
5	3.2	3.11 ± 0.12	1.55 ± 0.12	1.11 ± 0.07	1.42 ± 0.08	38 ± 12	*E*_3_/^4^*C*_1_
P	−7.6	3.38 ± 0.12	1.44 ± 0.10	2.19 ± 0.11	1.02 ± 0.04	30 ± 15	^4^*C*_1_

### Summary and Conclusions

By means of QM(DFT)/MM metadynamics we have unraveled the reaction mechanism of α-amylosucrase during hydrolysis, in particular the conformational dynamics of the glycosyl-enzyme intermediate (GEI), for which structural studies have assigned a ^4^*C*_1_ conformation. Our results show that the glycosylation reaction, assisted by the catalytic residues Asp286 (general base) and Glu328 (acid/base) features a dissociative transition state, in which the α-glucosyl residue at the *-1* subsite adopts a ^4^*H*_3_ conformation. The GEI intermediate adopts a ^4^*C*_1_ conformation, thus being not preactivated for catalysis. However, the active site is very dynamic and it can easily evolve toward another configuration, in which the *-1* sugar adopts a reactive *E*_3_ conformation, with a pseudo-axial orientation of the leaving group, as the catalytic water enters the active site. The two conformations can be viewed as two different states of the GEI intermediate, dry or wet. We conclude that the catalytic itinerary of amylosucrase for the Michaelis complex → TS → intermediate enzymatic half reaction could be either described indistinctly as ^4^*C*_1_ → [^4^*H*_3_]^‡^ → ^4^*C*_1_ (cyclic itinerary) or ^4^*C*_1_ → [^4^*H*_3_]^‡^ → *E*_3_ (linear itinerary). This reconciles the results obtained by X-ray, with an undistorted GEI (^4^*C*_1_), with the expected conformation of α-glucosidases (distorted GEI with a conformation near ^1^*S*_3_).

## Computational Details

### Model Building

The initial structure of the *Np*AS-sucrose complex was taken from the Michaelis complex structure of the Asp328Asn mutant (PDB 1JGI) (Mirza et al., 2001). The protonation states and hydrogen atom positions of all histidine residues were selected based on their hydrogen bond network (specifically, histidines 39, 173, 233, 332, 370, 392, 414, 463, 512, and 540 were considered neutral with the proton at N_ε_, histidines 192, 306, 565 and 591 were neutral with a proton at N_δ_ and histidines 377, 382, and 601 were protonated). All Asp and Glu residues were taken as deprotonated (i.e., negatively charged) except Glu328 (the acid/base residue). The system was solvated with 20725 water molecules and 16 sodium ions were added to achieve neutrality of the protein structure, forming a rectangular box with dimensions of 89.1 Å × 106.4 Å × 81.1 Å.

### Classical MD Simulations

Classical MD simulations of the *Np*AS-sucrose complex at room temperature were performed using the AMBER11 software (Case et al., 2010). The force-fields ff99SB (Hornak et al., 2006), GLYCAM06 (Kirschner et al., 2008) and TIP3P (Jorgensen et al., 1983) were used for the protein, sucrose substrate and solvent water, respectively. The system was equilibrated in several steps. First, all water molecules were relaxed with a gradient minimizer, holding the protein, and substrate fixed. Next, the whole system was allowed to relax. To gradually reach the desired temperature of 300 K, spatial constraints were initially added to the interactions between the protein and the substrate, while water molecules and sodium ions were allowed to move freely. The constraints were then removed and the whole system was allowed to reach the desired temperature. During all the process, the acid/base residue (Glu328) was very mobile, with the proton position evolving from cis to trans conformations and vice versa, thus its relative position with respect to the sugar was controlled with a smooth restraint (force constant of 5 kcal/mol/Å^2^). The simulation was pursued for 100 ps at constant pressure, allowing the cell volume to evolve, until the density stabilized (~1.06 g/cm^3^). The MD simulation was extended to 15 ns at constant volume without restraints until the system had reached equilibrium. A timestep of 1 fs was used, increasing it to 2 fs during the last 14 ns (using SHAKE). The −*1* sugar conformation evolved from ^4^*C*_1_ to *B*_3,O_ and returned to the original ^4^*C*_1_ conformation during the last 7 ns of the simulation. Analysis of the trajectories was carried out by using standard tools of AMBER and VMD (Humphrey et al., 1996). The root-mean-square-deviation (RMSD) of the protein backbone atoms with respect to the crystal structure was stabilized around 1.4 Å in the equilibrated structure. One snapshot from the last 0.5 ns of simulation was taken as starting structure for the subsequent QM/MM MD simulations.

### QM/MM MD Simulations

QM/MM MD simulations were performed with the CPMD software (Car and Parrinello, 1985), using the QM/MM interface developed by Laio et al. (2002). The QM region was considered as follows: (i) the whole sucrose molecule for the simulations of the conformational free energy landscape (FEL) of the *-1* sugar (α-glucosyl); (ii) the sucrose, the acid/base residue (Glu328, capped at the C_β_) and the nucleophile residue (Asp286, capped at the C_α_) for the simulation of the glycosylation reaction; (iii) same as (ii) plus the catalytic water, for the simulation of the deglycosylation reaction. In all cases, the frontier atoms between QM and MM region were described using pseudopotential carbon link atoms. The fictitious electronic mass of the Car-Parrinello Lagrangian was taken as 600 au and the timestep was set at 0.12 fs in all CPMD simulations. All systems were enclosed in an isolated cubic box of 12.0 Å × 12.0 Å × 12.0 Å, using a fictitious electron mass of 700 au and a time step of 0.12 fs. The Kohn-Sham orbitals were expanded in a plane wave (PW) basis set with a kinetic energy cutoff of 70 Ry. Ab initio pseudopotentials generated within the Troullier-Martins scheme were employed (Troullier and Martins, 1991). The Perdew, Burke and Ernzerhoff generalized gradient-corrected approximation (PBE) (Perdew et al., 1996) was selected in view of its good performance in previous work on isolated sugars (Biarnés et al., 2007; Marianski et al., 2016), glycosidases (Jin et al., 2016) and glycosyltransferases (Bilyard et al., 2018).

### QM/MM Metadynamics Simulations

QM/MM metadynamics (Laio and Parrinello, 2002; Barducci et al., 2011) simulations were performed to characterize the conformational FEL of the α-glucosyl residue of sucrose in the active site of *Np*AS and to simulate the different steps of the enzymatic reaction. The following collective variables were used: (i) Conformational FEL: the Cremer-Pople puckering coordinates (Cremer and Pople, 1975) phi and theta (φ, θ) of the α-glucosyl unit, following the methodology previously used in our group to rationalize and predict catalytic itineraries of GHs (Biarnés et al., 2007; Ardèvol and Rovira, 2015; Iglesias-Fernandez et al., 2015): (ii) glycosylation reaction: three collective variables representing the proton transfer (CV1), the nucleophilic attack (CV2) and the glycosidic bond cleavage (CV3) (Figure S3); (iii) deglycosylation reaction: three collective variables representing the covalent enzyme-substrate interaction (CV1), the water attack (CV2) and the proton transfer (CV3) were used (Figure S3). The metadynamics algorithm (Laio and Parrinello, 2002; Barducci et al., 2011), provided by the Plumed 2 plugin (Tribello et al., 2014), was used to explore the conformational free energy landscape of the systems. The height/width of the Gaussian terms was tested according to the oscillations of the CVs in free dynamics. In the case of the conformational FEL simulation, the height/width of the Gaussian terms was set at 0.75 kcal/mol/0.10 Å and a new Gaussian-like potential was added every 400 MD steps. The simulation of the conformational FEL was stopped once energy differences between the two wells (^4^*C*_1_ and *B*_3,O_) were maintained (1971 Gaussian terms were added), which was further tested by a time-independent free energy estimator (Tiwary and Parrinello, 2015). The error in the region of the two relevant minima and the pathway interconnecting them was ≤ 1.2 kcal/mol (Figure S6).

In the case of the glycosylation reaction, the height/width of the Gaussian terms was set at 1 kcal·mol^−1^/0.20 Å (CV1) and 1 kcal/mol/0.10 Å (CV2 and CV3) and a new Gaussian-like potential was added every 300 MD steps. Walls for each CV at appropriate distances were used to reduce the FEL space to the chemical event. For the deglycosylation reaction, values of 1 kcal/mol/ 0.10 Å (height/width) and 250 MD steps (deposition time) were used. Metadynamics simulations were stopped after one crossing over the transition state (Figure S7), as recommended for chemical reactions (Ensing et al., 2005). Previous work on carbohydrate-active enzymes shows that the error associated to the metadynamics is <1 kcal/mol using this criteria (Raich et al., 2016). The total number of Gaussian terms added was 2284 (glycosylation reaction) and 4947 (deglycosylation reaction). The reaction coordinate was taken from the minimum free energy pathway, computed according to the intrinsic reaction coordinate method (Fukui, 1981). Structures at a given point along the reaction coordinate were taken from averages over a small region defined by CV1 ± 0.2, CV2 ± 0.2, CV3 ± 0.2 Å and were used for analysis.

## Author Contributions

CR and IA designed calculations. SA-G performed and analyzed the simulations. JC helped with the computational work and analysis of the results. CR wrote the manuscript with the help of all authors.

### Conflict of Interest Statement

The authors declare that the research was conducted in the absence of any commercial or financial relationships that could be construed as a potential conflict of interest.
