# Supplementary material for: Conformational Itinerary of Sucrose During Hydrolysis by Retaining Amylosucrase
Source: Front Chem. 2019 Apr 30;7:269. doi: 10.3389/fchem.2019.00269 (PMC6502901; doi:10.3389/fchem.2019.00269)
Supplement: Supplementary file 1 [file Data_Sheet_1.pdf]

SUPPORTING INFORMATION

Conformational itinerary of sucrose during hydrolysis by retaining amylosucrase

Santiago Alonso-Gil[1], Joan Coines[1], Isabelle André[2,*], Carme Rovira[1,3,*]

[1]Departament de Química Inorgànica i Orgànica (Secció de Química Orgànica) & Institut de Quimica Teòrica i Computacional (IQTCUB), Universitat de Barcelona, Martí i Franquès 1, 08028 Barcelona, Spain.
[2] Laboratoire d'Ingénierie des Systèmes Biologiques et des Procédés, LISBP, Université de Toulouse, CNRS, INRA, INSA, Toulouse, France. 135, avenue de Rangueil, F-31077 Toulouse cedex 04, France.
[3]Institució Catalana de Recerca i Estudis Avançats (ICREA), 08020 Barcelona, Spain.

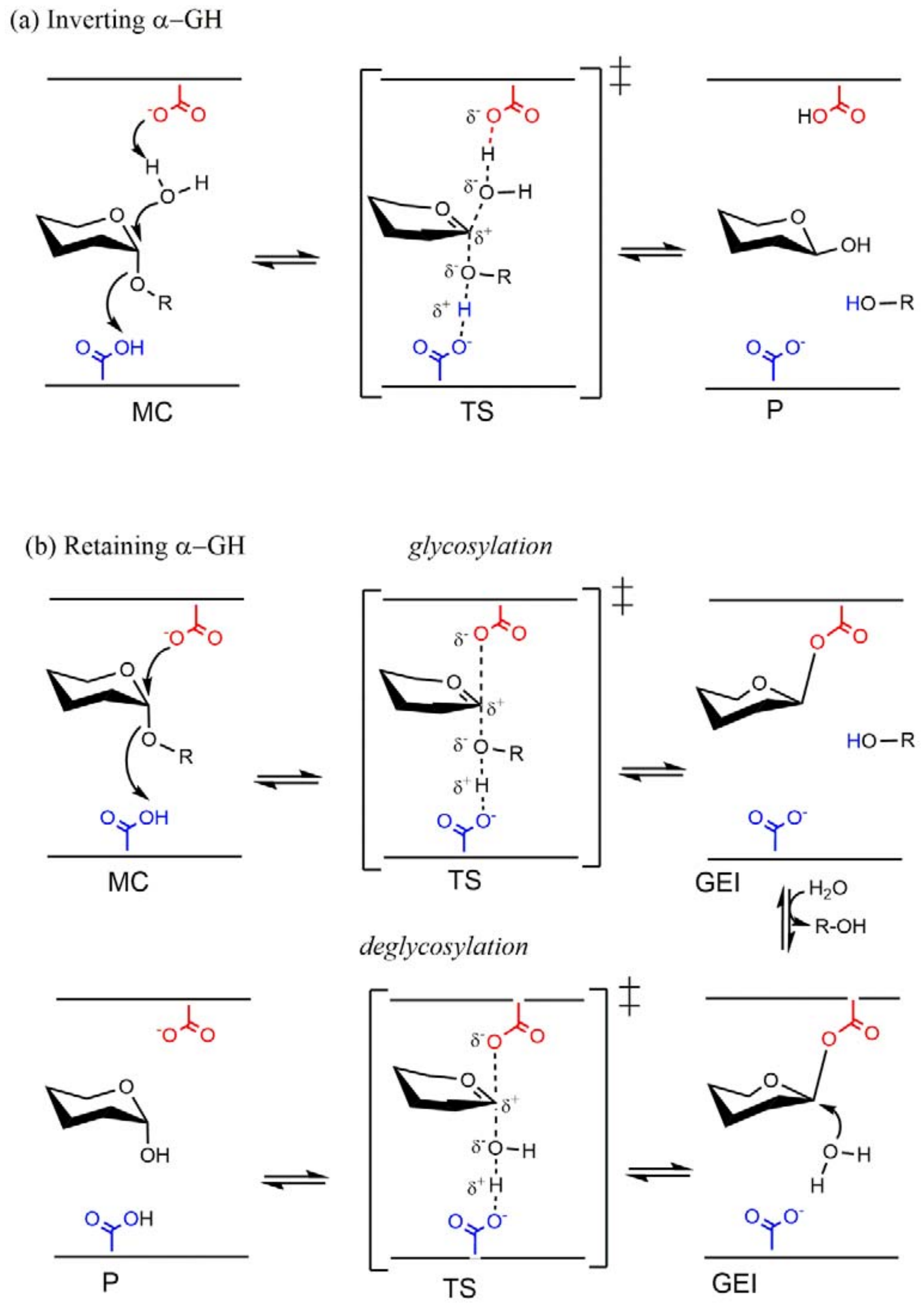

Figure S1. Generalized mechanisms for enzymatic glycoside hydrolysis in α-GHs: (a) inverting GHs and (b) retaining GHs. MC = Michaelis complex; TS = transition state; P = product complex.

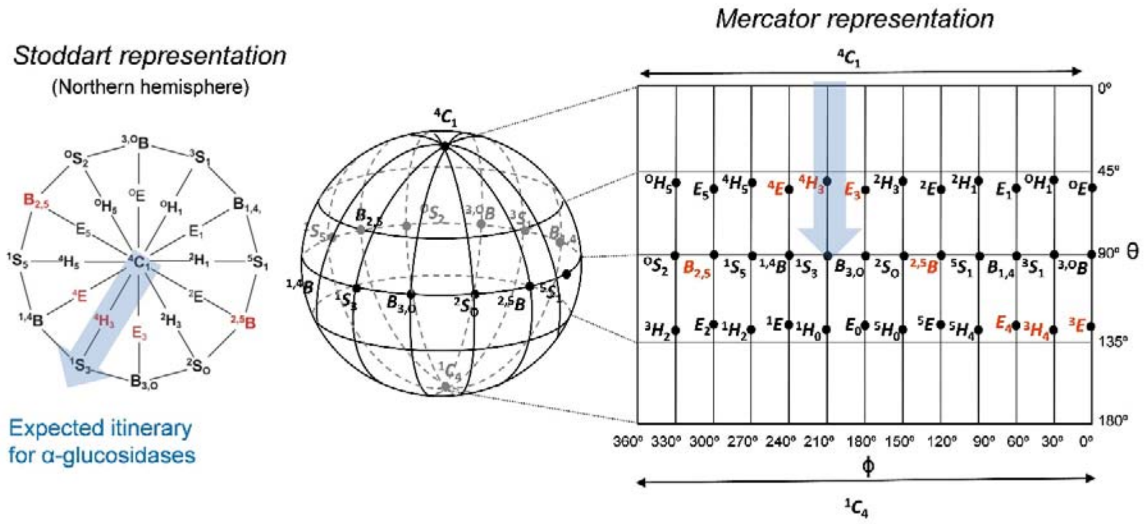

Figure S2. Stoddart (only the Northern projection is represented) and Mercator representations of the puckering sphere.

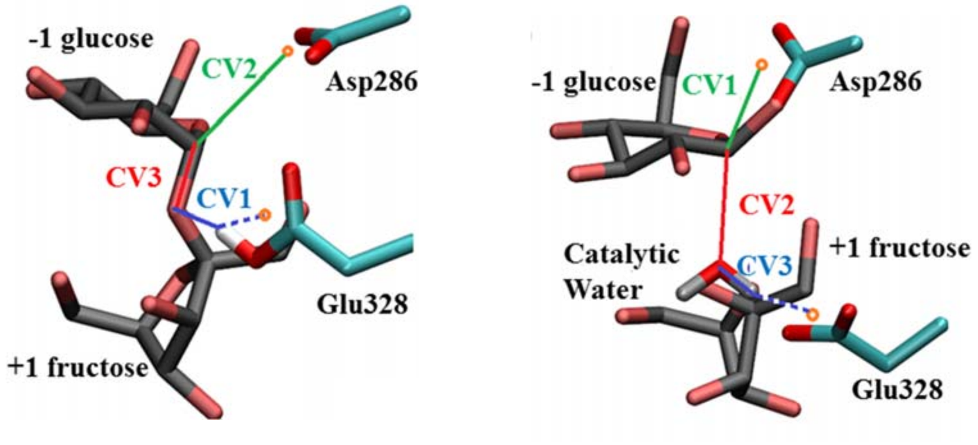

Figure S3. (a) QM region and collective variables (CVs) used to simulate the glycosylation reaction catalyzed by *Np*AS. CV1 (named as *proton transfer* variable) was taken as the distance difference between O-H (Glu328) and H···O1. To take into account that the proton can return to either of the two oxygens (which in fact happened during the metadynamics simulation), the center of mass of the CCO unit was considered instead of the position of the carboxylate oxygen. CV2 (named as *nucleophilic attack*) was taken as the distance between the nucleophile and the anomeric carbon (the center of mass of the CCO unit was considered). CV3 (*glycosidic bond cleavage*) is the C1-O1 distance. The orange dot represents the center of mass of the two oxygen atoms of a given carboxylate side chain. (b) QM region and CVs) used to simulate the deglycosylation reaction. Similar consideraton apply. CV1 = C1-O_{Asp286}, CV2 = C1-O_{wat} and CV3 = (O_{wat}-H) – (H-O_{Glu328}).

(a)

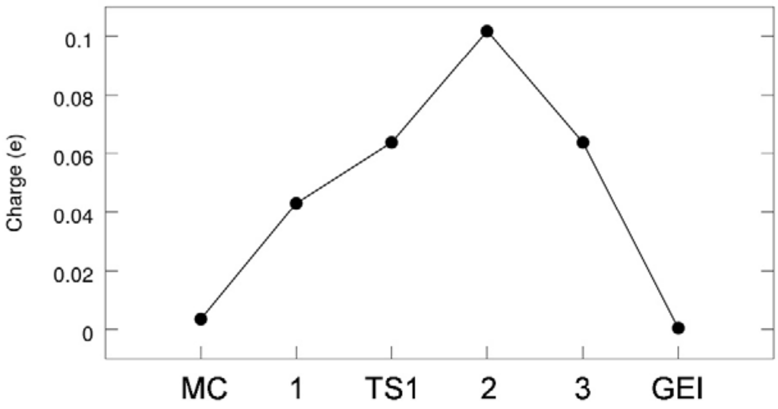

(b)

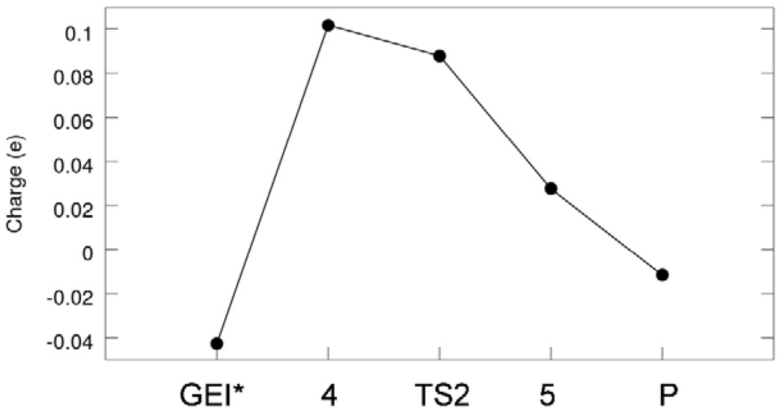

Figure S4. Evolution of the RESP charge of the anomeric center during the glycosylation and deglycosylation reactions (a and b, respectively).

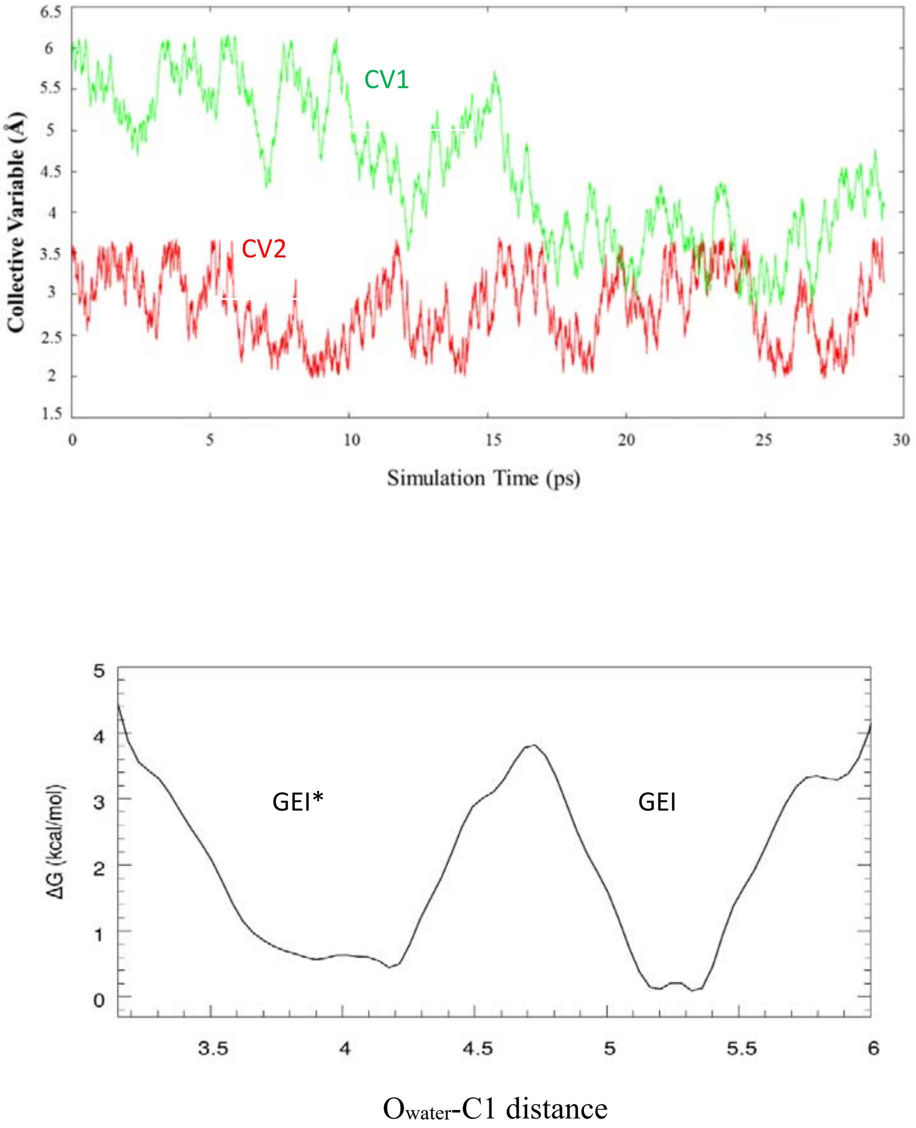

Figure S5. (a) Evolution of the two collective variables during the metadynamics simulation of the change from GEI to GEI*. CV1 is the distance between the water oxygen atom and the anomeric carbon (O_{water}-C1). CV2 is the hydrogen bond between Asp286 and the 6-OH. (b) Free energy change with respect to the approach of the water molecule to the anomeric center (free energy landscape projected into CV1).

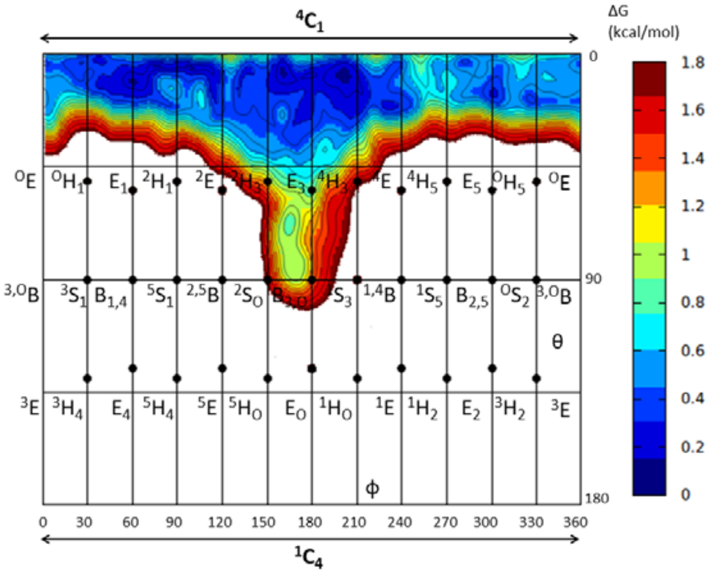

Figure S6. Error surface associated to the simulation of the conformational FEL of the *-1* sugar (α-glucosyl) of *Np*AS in complex with sucrose, according to the time-independent free energy estimator described by Tiwary and Parrinello (*J. Phys. Chem. B*. 2015, 119, 736-742).

(a)

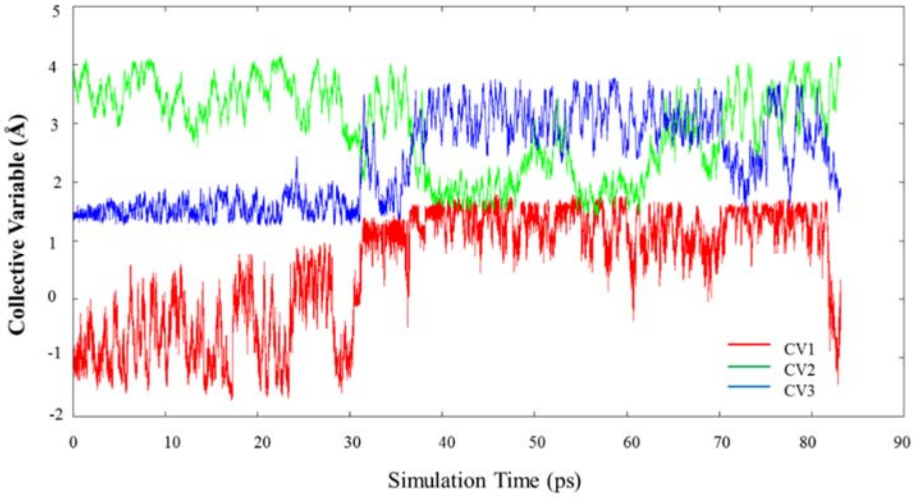

(b)

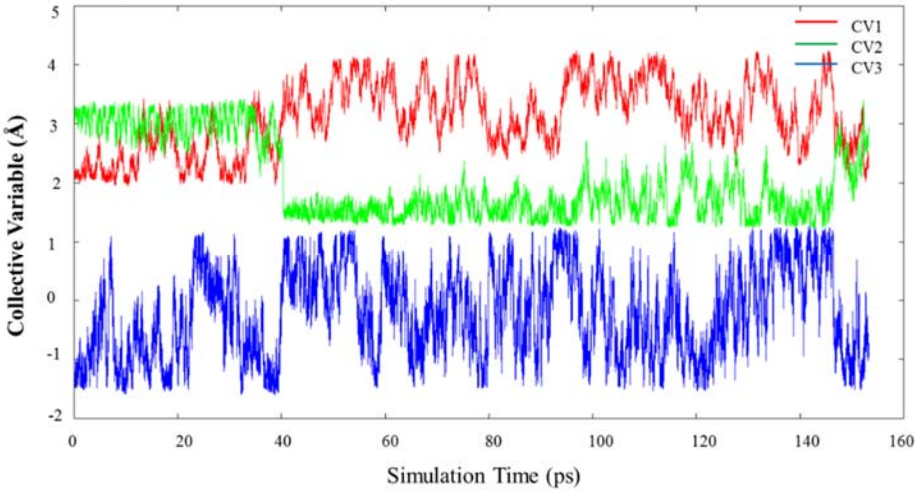

Figure S7. Evolution of the three collective variables (see Figure S3) during the metadynamics simulation of the glycosylation reaction (a) and deglycosylation reaction (b).
